# Novel KDM2B/SAV1 Signaling Pathway Promotes the Progression of Gastric Cancer

**DOI:** 10.1155/2023/1230182

**Published:** 2023-03-31

**Authors:** Ning Li, Haifei Song, Zhiqin Chen, Chen Chen, Ming Quan

**Affiliations:** ^1^Department of Oncology, Shanghai General Hospital, Shanghai Jiao Tong University School of Medicine, Shanghai, China; ^2^Department of Oncology, Shanghai East Hospital, Tongji University School of Medicine, Shanghai, China

## Abstract

Salvador homologue 1 (SAV1), which is reported to act as a tumor suppressor in different types of cancer, is one of the key components of the Hippo pathway. However, the expression and mechanisms of SAV1 in the development and progression of gastric cancer (GC) remain to be elucidated. Immunohistochemistry (IHC) was performed in the present study to assess the expression levels of SAV1 and lysine-specific demethylase 2B (KDM2B) in GC tissues. The biological effects of SAV1 on GC cell proliferation, migration, and invasion were studied *in vitro*. KDM2B transcriptionally regulates SAV1 expression in several GC cell lines, and molecular experiments were performed to investigate underlying mechanisms. The expression level of SAV1 was significantly decreased in GC tissues and cell lines, negatively associated with tumor invasion depth, lymph node metastasis, and TNM stage, and positively associated with the overall survival of patients with GC. SAV1 overexpression inhibited the proliferation, migration, and invasion of GC cells. Further mechanistic studies revealed that KDM2B transcriptionally regulated SAV1 expression and further regulated the Hippo signaling pathway. To conclude, the present study demonstrated that KDM2B transcriptionally regulated SAV1 expression and promoted GC progression.

## 1. Introduction

Gastric cancer (GC) remains a prevalent disease worldwide. GC resulted in ∼783,000 deaths in 2018, making it the third leading cause of cancer-related deaths worldwide. The incidence rates of GC are significantly increased in Eastern Asia, especially in Japan, South Korea, and China [1]. In China, GC ranks third in annual morbidity causes and mortality causes [2]. Most patients are already at the late stages at the time of diagnosis, and the 5-year survival rate of advanced GC is ∼5.2%; hence, early detection of GC is crucial [3]. Although medical technology has improved, the overall diagnostic rate remains low. Therefore, it is important to study the mechanisms that promote the development and progression of GC and identify novel targets to improve therapeutic effects and prognosis.

Salvador adaptor protein (SAV), which contains two protein-protein interaction modules known as WW domains, is considered to function as a scaffolding protein for the mammalian Hippo pathway [4]. Salvador homologue 1 (SAV1), also known as WW45, is the human homolog of Salvador that couples mammalian Ste20-like 1 and 2 kinases (MST1/2) to large tumor suppressor kinases 1 and 2 (LATS1/2) to form the Hippo signaling pathway [5]. As an adaptor protein, SAV1 acts as a coactivator of MST1/2 kinases and can directly bind to MST1/2 and induce the kinase cascade via promoting the phosphorylation of MST 1/2, LATS 1/2, yes-associated protein (YAP), and/or transcriptional coactivation with PDZ-binding motif (TAZ). The phosphorylation of YAP and TAZ can lead to their cytoplasmic translocation, ubiquitination, and degradation. The cytoplasmic translocation of YAP/TAZ inhibits the transcription of their downstream target oncogenes and leads to tumor suppression [6, 7]. SAV1 acts as a tumor suppressor in different types of cancer, including pancreatic [8], colon [9], and lung cancer [10]. SAV1 downregulation induces tumorigenesis and metastasis and is closely associated with a poor prognosis of lung and pancreatic cancer [8, 10]. However, the roles and mechanisms of SAV1 expression and function in GC remain to be clarified.

Histone demethylase lysine-specific demethylase 2B (KDM2B) plays a role in numerous cellular processes, including cell differentiation, senescence, and the self-renewal of stem cells. It was recently revealed that KDM2B expression was increased in different cancer types and acts as an epigenetic regulator in cancer development and progression. KDM2B (also known as JHDM1B, Ndy1, and FBXL10) regulates the demethylation of H3K36me2 and the expression of a series of genes at the transcriptional level. In pancreatic ductal adenocarcinoma (PDAC), KDM2B interacts with KRAS-G12D to promote tumorigenesis in mouse models [11]. Our previous studies demonstrated that KDM2B expression was increased in PDAC and promoted PDAC progression via the Hippo pathway by transcriptionally regulating MOB kinase activator 1A expression [12]. Another study showed that KDM2B regulated cell adhesion and migration of prostate cancer cells [13]. However, the roles and mechanisms of KDM2B in promoting GC progression remain to be further studied.

The present study investigated the roles, expression, and regulatory mechanisms of SAV1 in GC. The present study revealed that increased SAV1 expression decreased the growth and metastasis of GC. Mechanistic studies showed that KDM2B could directly bind to the promoter region of the SAV1 gene, which resulted in the methylation of H3K27 and the decreased transcription and expression of SAV1, further resulting in GC progression via the Hippo signaling pathway.

## 2. Materials and Methods

### 2.1. Human Tissue Microarrays (TMAs) and IHC

The protein expression of SAV1 and KDM2B was tested with human tissue microarrays (TMAs) which were bought from Shanghai Outdo Biotech Company (China). Totally, the TMA contains 100 primary GC tissues and 80 adjacent normal gastric tissues. Clinical and demographic information, including gender, age, TNM stages, differentiation, and overall survival from the time of diagnosis, was available. Immunohistochemical analysis was conducted with anti-SAV1 (HPA0018085, Sigma-Aldrich, diluted 1 : 100) and anti-KDM2B (SAB2702002, Sigma-Aldrich, diluted 1 : 300). Then, the immunostaining signals of SAV1 and KDM2B were evaluated by at least two pathologists who were blinded to the clinical information. The percentage of SAV1- or KDM2B-positive cells was divided into four groups: 1 was <25%, 2 was from 25% to 50%, 3 was from 50% to 75%, and 4 was >75%. The staining intensity of SAV1- or KDM2B-positive cells was scored into four categories: 0 was absent, 1 was weak infiltration, 2 was moderate infiltration, and 3 was strong infiltration. The final score was the result of multiplying the intensity and the percentage. In statistical analyses, the expression of SAV1 and KDM2B was further divided into low SAV1 or KDM2B expression which was from 0 to 4 or high SAV1 or KDM2B expression which was from 6 to 12.

### 2.2. Cell Lines

The human GC cell lines, namely, HTB103, SNU-1, AGS, and NCI-N87, were obtained from the American Type Culture Collection. The TMK-1 cell line was obtained from Masashi Kanai (Kyoto University, Kyoto, Japan), and the SK-GT5 cell line was obtained from Gary K. Schwartz (Memorial Sloan-Kettering Center, New York, NY).

### 2.3. Plasmids and Short Hairpin RNAs (shRNAs)

The p2xFlag-CMV2 SAV1 (pSAV1) plasmid was obtained from Addgene (Plasmid #18970) [14]. Full-length SAV1 was amplified and cleaved as a BamHI-EcoRI fragment and then cloned into the retroviral expression vector (pBABEpuro). The retroviruses were produced and stored as previously reported [12]. The amplified DNA fragments of the inserts and flanking regions of the plasmids were all verified by sequencing. A 900 bp fragment containing SAV1 transcriptional start sites (TSSs) was amplified and subcloned into the pGL3-basic vector (Promega) (pGL3-SAV1). The primers were as follows: 5′-AGC TGG TAC CTC CCT GAT ACT CAG TAG AGG ATC-3′ (forward) and 5′-AGC TAA GCT TTC TTT CGG GAC AGC ATC CTT CT-3′ (reverse). shRNA is constructed with the target sequences of SAV1 sequence 1 : 5′-TGC AGA AAT TCC TGA CTG GCT TCA GGT TT-3′ and sequence 2 : 5′-CCT GTG AAA TAT GAC CAC ATT CTG AAG TG-3′ [4], and shRNA sequences which target KDM2B were reported previously [14].

### 2.4. Dual-Luciferase Reporter Assay

GC cells were cotransfected with indicated vectors or a control vector, pGL3-SAV1, and the *β*-actin/Renilla luciferase reporter. After transfecting for 24 hours in each group, we used the dual luciferase assay system (Promega) to test the luciferase activity in each group cells.

### 2.5. Chromatin Immunoprecipitation Assay

The chromatin immunoprecipitation (ChIP) assay kit (Millipore Technology, Billerica, MA) was used to perform the ChIP assay. According to the manufacturer's protocol, 2 × 10^6^ tumor cells were prepared, and the anti-KDM2B antibody was purchased from Millipore (#09–864). The resulting DNA samples were tested using quantitative real-time PCR. The primers for qPCR were as follows: 5′-ATC TGC GTC GAG CTT CCC AGA ATT-3′ (forward) and 5′-ATT CCT TCT TCA CGT ACT TCC CCT-3′ (reverse). In qPCR, an 80 bp region of the SAV1 promoter was amplified and analyzed.

### 2.6. Gene Transfection

In transient transfection, GC cells were plated into six-well or twenty-four-well plates. Lipofectamine LTX (Invitrogen) and Lipofectamine 2000 CD (Invitrogen) were used to transfect the plasmids or shRNAs. After 48 hours, the functional assays were performed. For retroviral transduction, the GC cells were plated into six-well plates for 24 hours and the confluency was about 50%. A mixture of retroviruses and hexadimethrine bromide (Polybrene; 5 *μ*g/mL) were used to infect the GC cells, and puromycin with a concentration of 2 *μ*g/mL was used to select stable populations.

### 2.7. Quantitative Real-Time RT-PCR

The RNA expression levels of SAV1 were analyzed using quantitative real-time RT-PCR. The SYBR Green reagent with an ABI Prism 7000HT sequence detection system (Applied Biosystems) was used. The sequences of the PCR primers were as follows: SAV1, 5′-GCA GGG GAA GTA CGT GAA GA-3′ (forward) and 5′-GCA TTA GGG CTT GAA TCT GG-3′ (reverse); *β*-actin, 5′-AGC CGG GCT CTT GCC AAT-3′ (forward) and 5′-AGT TAG CGC CCA AAG GAC CA-3′ (reverse) [12].

### 2.8. Western Blot Analysis

Total cell lysates were extracted. Western blotting was performed using total cell lysates. The primary antibodies used in western blot were anti-SAV1 (HPA0018085, Sigma), anti-YAP (#12395, Cell Signaling Technology), anti-KDM2B (SAB2702002, Sigma), anti-pYAP (#13008, Ser127, Cell Signaling Technology), anti-H3K27me3 (#9733, Cell Signaling Technology), and anti-CTGF (#86641, Cell Signaling Technology). Anti-actin (rabbit; Santa Cruz Biotechnology) was used as equal protein-sample loading. Anti-mouse IgG, anti-goat IgG, or anti-rabbit IgG (Santa Cruz Biotechnology) were used as secondary antibodies. Quantity One analysis software was used to quantify the bands (version 4.6; Bio-Rad).

### 2.9. Colony Formation and Spheroid Colony Formation Assay

24-well plates were seeded with two hundred cells from each group as indicated. The cells were allowed to grow for two weeks in the medium which was changed twice a week. After two weeks, 4% paraformaldehyde was used to fix the cells and 0.1% crystal violet solution was used to stain the cells for 10 minutes. A microscope at 40x magnification was used to count the colonies (>20 cells). The percentage of the control was the result of the test.

The GC cells were infected with pBABE-SAV1 or pBABEpuro and were maintained in the DMEM/F12 medium supplemented with bFGF (20 ng/ml) and EGF (20 ng/ml) and B27 supplements (Invitrogen), and the spheroids were counted. All experiments were performed in triplicate and repeated twice.

### 2.10. Cell Migration and Invasion

Cell migration and invasion assays were conducted using modified 24-well Boyden chambers which were purchased from BD Biosciences and were used to test migration and invasion abilities of GC cells. Briefly, cells from different groups were treated as indicated for 24 hours. Then, the cells were suspended in DMEM at a concentration of 8 × 10^4^/ml. The cells were prepared in 500 *μ*l of DMEM and were loaded in the upper wells. The medium, containing 20% FBS, was used as a chemoattractant stimulus in the lower wells. The cells which migrated on the bottom surface of the filter were fixed and stained with H&E. The migrated cells, in three randomly selected fields, were counted under a microscope at a magnification of 200x.

### 2.11. Statistical Analysis

The correlation of the expression of SAV1 and KDM2B in the TMA was analyzed using the Spearman rank correlation coefficients. The significance of differences among the covariates in TMA was determined with a two-tailed*X*^2^ test or Fisher exact test. The Kaplan–Meier method was used to estimate OS, and the log-rank test was used to compare the differences. Multivariate analysis was used to analyze the significant variables for independent prognosis. All the *in vitro* experiments were performed at least twice, and one representative result of the two or three experiments with similar results was presented. The significance of the results of the *in vitro* experiments was analyzed with Student's *t-test* (two-tailed) or one-way analysis of variance. A *P* value less than 0.05 was considered to be statistically significant. The statistical analysis was performed via the SPSS software program (version 13.0; IBM Corporation).

## 3. Results

### 3.1. SAV1 Expression Is Directly Associated with Pathological Features of GC

**Figure 1:**
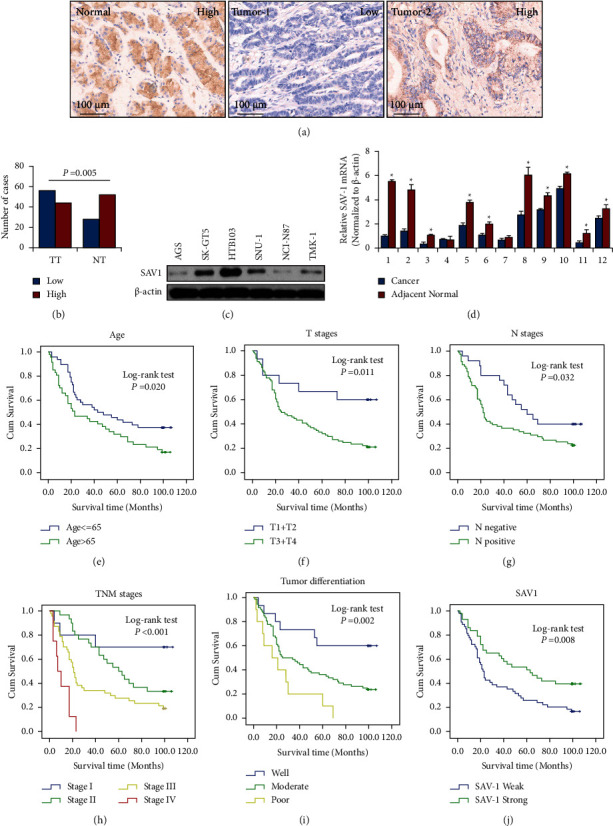
SAV1 expression and its correlation with the clinicopathological features of GC. Tissue microarray GC specimens were immunostained with a specific anti-SAV1 antibody. (a) Representative images of SAV1 expression in adjacent normal gastric tissues and GC tissues. Markedly high SAV1 expression was observed in adjacent normal tissue (normal, left panel), low SAV1 expression was observed in tumor tissue (tumor 1, middle panel), and high SAV1 expression was observed in tumor tissue (tumor 2, right panel). (b) SAV1 expression was significantly lower in tumors (TT) than that in adjacent normal tissue (TN). (c) SAV1 protein levels in GC cell lines. (d) The mRNA levels of SAV1 in 12 paired GC and adjacent normal gastric tissues were measured using reverse transcription-quantitative PCR. (e) Age of >65 years (*P*=0.020), (f) invasion depth (*P*=0.011), (g) lymph node metastasis (*P*=0.032), (h) clinical stage (TNM stage) (*P* < 0.001), and (i) poorer tumor differentiation (*P*=0.002) were negatively associated with the OS of patients with GC. (j) SAV1 expression was positively associated with the OS of patients with GC (*P*=0.008). SAV1, Salvador homologue 1; GC, gastric cancer; OS, overall survival.

To determine the roles of SAV1 in GC pathogenesis, the present study first analyzed SAV1 expression in GC tissue arrays by IHC. The clinicopathological characteristics of the TMA are shown in Table S1. SAV1-positive staining was mainly observed in the cytoplasm of adjacent normal gastric tissue and several GC cancer tissues. SAV1 expression in cancer tissues was much lower than that in tumor-adjacent normal gastric tissues (Figures 1(a) and 1(b)). Decreased SAV1 expression was positively associated with tumor invasion depth (T stage; Table S1), lymph node metastasis (N stage; Table S1), and TNM stages (Table S1). The prognostic value of SAV1 and classical clinicopathological characteristics on patient survival was determined by the Kaplan–Meier analysis and log-rank test. Univariate analysis showed that SAV1 expression was associated with the OS of patients with GC (*P* < 0.001; Figure 1(j) and Table S3). Univariate analysis also indicated that age (*P* < 0.001; Figure 1(e) and Table S3), *T* stages (*P*=0.011; Figure 1(f) and Table S3), *N* stages (*P*=0.032; Figure 1(g) and Table S3), TNM stages (*P* < 0.001; Figure 1(h) and Table S3), and tumor differentiation (*P*=0.002; Figure 1(i) and Table S3) were correlated with patient OS. Furthermore, multivariate analysis showed that age (*P*=0.021; Table S3), tumor differentiation (*P*=0.024; Table S3), and TNM stages (*P*=0.008; Table S3) were independent prognostic factors for patients with GC.

The present study further assessed SAV1 expression in GC cell lines via western blotting. SAV1 levels were significantly lower in most cancer cell lines (Figure 1(c)). The present study then analyzed the mRNA levels of SAV1 from 12 paired GC and adjacent normal gastric tissues using qPCR. The results showed that the mRNA levels of SAV1 were significantly decreased in GC tissues compared with adjacent normal tissues (*P* < 0.05; Figure 1(d)), indicating that SAV1 downregulation may be involved in GC pathogenesis.

### 3.2. SAV1 Inhibits GC Cell Proliferation, Migration, and Invasion *In Vitro*

**Figure 2:**
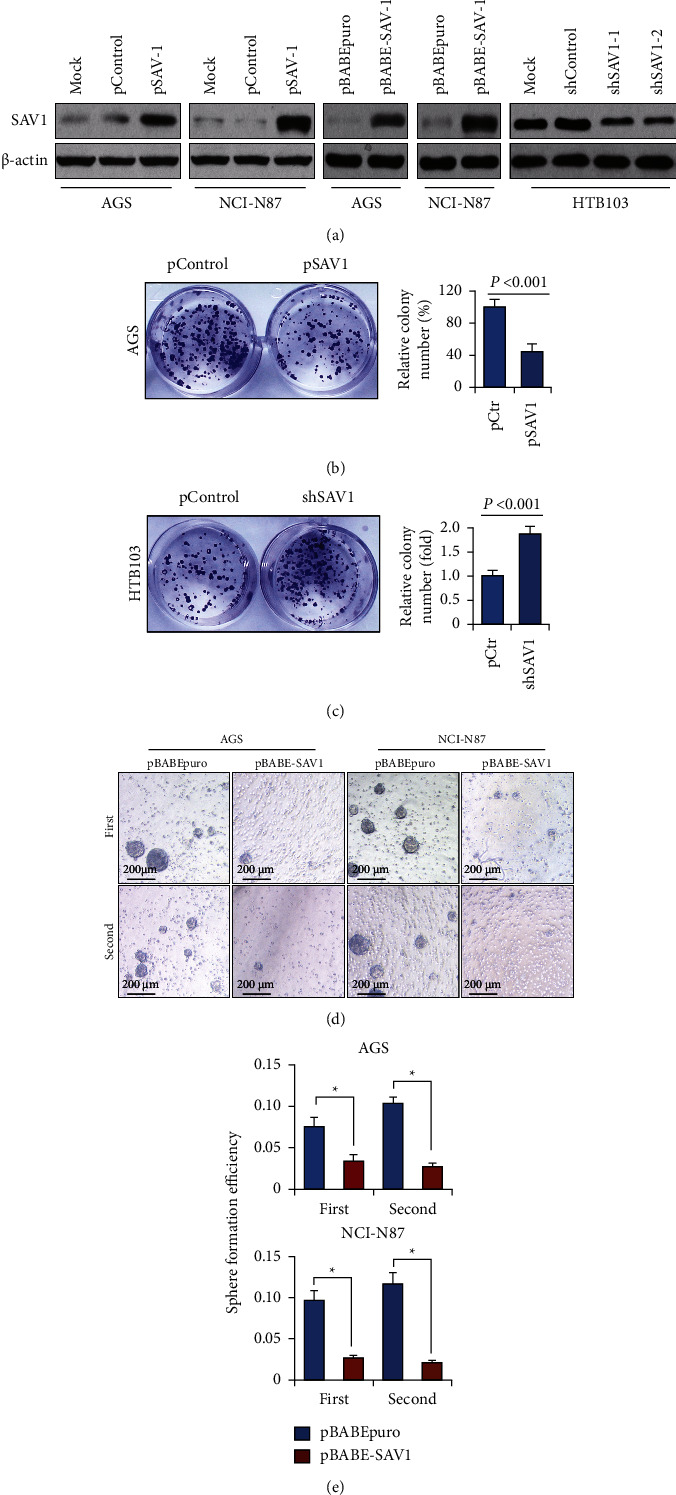
SAV1 suppresses GC cell proliferation *in vitro*. (a) Transfection efficiency of SAV1 overexpression vectors and shRNA in GC cell lines determined via western blotting. AGS and NCI-N87 cells were transfected with pSAV1 or control vectors. HTB103 cells were transfected with shSAV1-1, shSAV1-2, or control shRNAs. AGS and NCI-N87 cells were infected with retroviruses containing SAV1 (pBABE-SAV1) and empty retroviral expression vector (pBABE-puro). (b, c) The colony formation assay was performed on AGS and HTB103 cells in 24-well plates, and the numbers of colonies were counted after 14 days. (d, e) The spheroid colony formation assay was performed on AGS and NCI-N87 cells. SAV1 overexpression decreased the number and size of the first and second generations of spheroids. SAV1, Salvador homologue 1; GC, gastric cancer; shRNA, short hairpin RNA.

To investigate the biological roles of SAV1 in GC, AGS and NCI-N87 cells (which express low levels of endogenous SAV1) were transfected or infected with SAV1 expression vectors (AGS/NCI-N87-pSAV1 and AGS/NCI-N87-pBABE-SAV1). Empty expression vectors were used as the control (AGS/NCI-N87-Control and AGS/NCI-N87-pBABEpuro). Infected cells were selected using puromycin, and it was found that pooled drug-resistant cells had significantly elevated SAV1 expression (Figure 2(a)). HTB103 cells (which express higher levels of endogenous SAV1) were transfected with shSAV1-1, shSAV1-2, and control vectors. The protein levels of SAV1 in these cells were measured using western blotting (Figure 2(a)). Western blotting revealed that SAV1 levels were significantly overexpressed in AGS and NCI-N87 cells. shSAV1-2 resulted in significantly lower expression of SAV1 compared with shSAV1-1. Hence, shSAV1-2 was chosen as shSAV1 for subsequent experiments.

To investigate the roles of restored SAV1 expression in GC cell proliferation, AGS and NCI-N87 cells were used for colony formation assays. As shown in Figures 2(b) and 2(c), restoration of SAV1 expression significantly suppressed colony formation in AGS cells, but SAV1 knockdown promoted colony formation in HTB103 cells. Furthermore, the role of SAV1 in GC cell spheroid formation was assessed. Restored SAV1 expression significantly reduced spheroid numbers and sizes in the first and second generations of AGS and NCI-N87 cells (Figures 2(d) and 2(e)). These results revealed the suppressive roles of SAV1 in GC cell proliferation.

**Figure 3:**
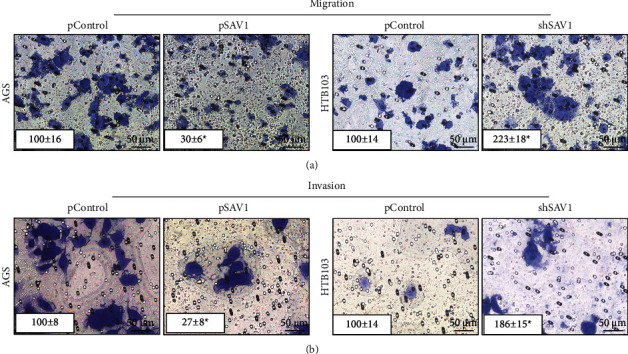
SAV1 inhibits gastric cancer cell migration and invasion *in vitro.* (a, b) AGS cells were transfected with pSAV1 or control vectors, and HTB103 cells were transfected with shSAV1 or control vectors. The migration and invasion of AGS and HTB103 cells were determined. The representative migrated or invaded tumor cells were photographed. Data represent the mean ± SD of triplicates. SAV1, Salvador homologue 1; shRNA, short hairpin RNA.

To further assess the effects of restored SAV1 expression on the migration and invasion of GC cells, AGS and HTB103 cells were transfected with pSAV1 or shSAV1, respectively. Similarly, restored SAV1 expression suppressed the migration and invasion of AGS cells, but SAV1 knockdown promoted the migration and invasion of HTB103 cells (Figures 3(a) and 3(b)). Collectively, these data demonstrated that SAV1 served as a tumor suppressor by inhibiting the proliferation, migration, and invasion of GC cells.

### 3.3. KDM2B Transcriptionally Inhibits SAV1 Expression

**Figure 4:**
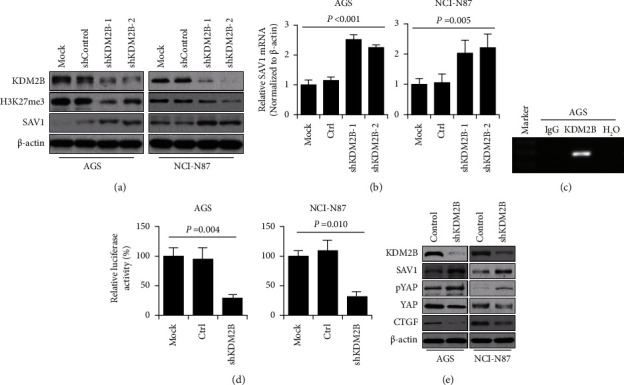
KDM2B transcriptionally regulates SAV1 expression. (a) KDM2B shRNAs and control vectors were transfected with AGS and NCI-N87 cells. Western blotting was used to analyze the expression of KDM2B, H3K27me3, and SAV1. *β*-Actin acted as the internal control. (b) AGS and NCI-N87 cells were transfected with KDM2B shRNAs and control vectors. Reverse transcription-quantitative PCR was performed to analyze the mRNA levels of SAV1. (c) Chromatin was isolated from AGS cells. The binding of KDM2B and negative control water to the SAV1 promoter was determined using chromatin immunoprecipitation. (d) Constructions of SAV1 promoter reporters. AGS and NCI-N87 cells were cotransfected with 0.2 *µ*g SAV1 promoter luciferase constructs pLuc-SAV1 and 0.2 *µ*g KDM2B shRNA or control vectors. Promoter activity was examined using the dual luciferase assay kit. ^*∗*^*P* < 0.05. (e) AGS and NCI-N87 cells were transfected with shKDM2B and control vectors. The levels of SAV1, p-YAP, YAP, and CTGF were analyzed via western blotting. *β*-Actin acted as the internal control. SAV1, Salvador homologue 1; shRNA, short hairpin RNA; KDM2B, lysine-specific demethylase 2B; YAP, yes-associated protein; CTGF, connective tissue growth factor; p phosphorylated.

SAV1 expression is modulated by hypermethylation in PDAC [8]. However, the mechanism of decreased SAV1 expression in GC has not been demonstrated. Tzatsos et al. reported that KDM2B binds to TSS, resulting in decreased H3K27me2 and suppressing the expression of a series of genes involved in development [11]. The present study then assessed whether KDM2B regulated SAV1 expression. KDM2B was knocked down by shKDM2B-1 and shKDM2B-2 in AGS and NCI-N87 cells. Both shKDM2B-1 and shKDM2B-2 could effectively knock down KDM2B, with shKDM2B-2 showing more effective knockdown (Figure 4(a)). shKDM2B-2 was then used as shKDM2B in subsequent experiments. Western blotting revealed that KDM2B knockdown led to decreased H3K27me3 levels (Figure 4(a)). In addition, KDM2B knockdown increased both the mRNA and protein levels of SAV1 (Figures 4(a) and 4(b)). ChIP revealed that KDM2B directly bound to the TSS region of SAV1 in AGS cells (Figures 4(c)). The SAV1 promoter reporter (pLuc-SAV1) was then generated, which contained the surrounding bases of TSS. The luciferase assay results showed that KDM2B knockdown significantly elevated the transcriptional activity of the SAV1 promoter reporter (Figure 4(d)). The aforementioned data demonstrated that SAV1 was a direct downstream target of KDM2B and that KDM2B transcriptionally regulated SAV1 expression.

SAV1 is the core component of the Hippo signaling pathway and suppresses the oncogenic transcriptional module, YAP, which functions as transcriptional coactivators and promotes GC progression [6, 7]. KDM2B knockdown led to increased levels of SAV1 protein and YAP phosphorylation and decreased protein levels of YAP and its typical downstream target CTGF (Figure 4(e)). These results demonstrated that KDM2B regulated the Hippo pathway via transcriptionally regulating SAV1.

### 3.4. KDM2B Expression Is Correlated with Pathological Features of GC and Negatively Associated with SAV1

**Figure 5:**
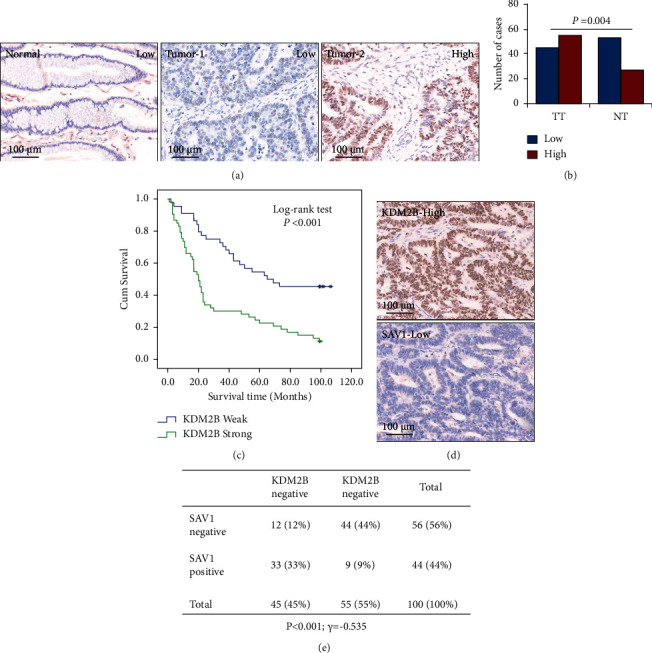
KDM2B expression is associated with GC progression and SAV1. TMA GC specimens were immunostained with a specific anti-KDM2B antibody. (a) Representative images of KDM2B expression in GC specimens and adjacent normal gastric tissue specimens. Markedly low KDM2B expression was observed in adjacent normal tissue (left panel), low KDM2B expression was observed in tumor tissue (middle panel), and high KDM2B expression was observed in tumor tissue (right panel). (b) KDM2B expression was significantly higher in tumors (TT) than that in adjacent normal tissue (TN). (c) KDM2B expression was negatively associated with the OS of patients with GC. (d) Representative images of high KDM2B expression and low SAV1 expression in TMA sections. (e) KDM2B and SAV1 protein expression in TMA sections from the cohort; the negative correlation between KDM2B and SAV1 expression was determined using Pearson's correlation coefficient analysis. N = 100. R = −0.535. *P* < 0.001. KDM2B, lysine-specific demethylase 2B; GC, gastric cancer; SAV1, Salvador homologue 1; OS, overall survival; TMA, tissue microarray.

The present study provided evidence that KDM2B transcriptionally suppressed SAV1 expression. To further confirm the results, the protein levels of KDM2B in the serial GC tissue array of SAV1 were analyzed using IHC. KDM2B was mainly positively stained in the nuclei of cancer tissues and was more highly expressed in cancer tissues than in tumor-adjacent normal gastric tissues (Figures 5(a) and 5(b)). Furthermore, KDM2B expression was positively associated with *T* stages (*P*=0.001; Table S2), lymph node metastasis (*P* < 0.001; Table S2), and higher TNM stages (*P* < 0.001; Table S2). The prognostic value of KDM2B expression on GC patient survival was tested using the Kaplan–Meier analysis and log-rank tests. Univariate analysis showed that KDM2B was negatively associated with the OS of patients with GC (*P* < 0.001; Figure 5(c) and Table S3). However, multivariate analysis showed that KDM2B was not an independent prognostic factor for GC (*P*=0.446; Table S3). The present study then further analyzed the correlation between SAV1 and KDM2B expression within the same cohort. As shown in Figures 5(d) and 5(e), a direct negative correlation between SAV1 and KDM2B expression was found in GC tissues (*r* = −0.535; *P* < 0.001). These data further confirmed that KDM2B is a negative regulator of SAV1.

## 4. Discussion

SAV1 is one of the key components of the Hippo signaling pathway and acts as a coactivator of MST1/2 kinases by directly binding to MST 1/2 and promoting the phosphorylation of MST 1/2, LATS1/2, YAP, and TAZ. The Hippo signaling pathway was reported to play essential roles in the development and progression of cancer and is considered a potential therapeutic target [15]. Thus, the roles of SAV1 in cancer have gained increasing attention. It was reported that SAV1 repressed the growth of colorectal and pancreatic cancer [9]. In glioblastoma, repressed SAV1 expression promoted the stem cell phenotype of glioblastoma cells [16]. In another study, deletion of both PTEN and SAV1 in the liver promoted the development of liver cancer in mice [17]. In addition, SAV1 plays an important role in the chemosensitivity of ovarian cancer cells to cisplatin [18]. However, the roles of SAV1 in GC have not been identified. The present study provided four lines of evidence to demonstrate the tumor suppressor role of SAV in GC. First, the present results showed that SAV1 expression was decreased in GC cell lines and tissues. Second, SAV1 expression was negatively associated with tumor invasion depth, lymph node metastasis, TNM stages, and patient survival. Third, restored expression of SAV1 suppressed the proliferation and cell spheroid formation of GC *in vitro*. Moreover, SAV1 knockdown promoted colony formation and cell spheroid formation of GC cells. Fourth, SAV1 overexpression inhibited GC cell migration and invasion *in vitro*, but SAV1 knockdown promoted the migration and invasion of GC cells. Collectively, these findings demonstrated that SAV1 suppressed the proliferation, migration, and invasion of GC cells and also functioned as a tumor suppressor in GC. SAV1 expression was reported to be decreased in different types of cancer, including colon, lung, renal cell, liver, and pancreatic cancer [8, 9, 19–21]. It was reported that microRNA (miRNA)-21, miRNA-181c, miR-149-5p, miRNA-130b, long noncoding HOTAIR, and hypermethylation of the DNA promoter region modulated the expression of SAV1 [8, 9, 16, 18, 22, 23]. In another study, MST2 was found to be coexpressed with SAV1, phosphorylated SAV1 at Thr-26, Ser-27, Ser-36, and Ser-269, and promoted cell death [24]. However, the mechanism of suppressed SAV1 expression requires further study. A study on pancreatic cancer showed that KDM2B bound to TSS, decreased the levels of H3K27me3, and regulated the expression of a series of genes, and ChIP-sequencing results showed that SAV1 is one of the potential target genes of KDM2B [11]. The present study then further determined whether KDM2B modulated SAV1 expression. The results showed that KDM2B knockdown increased both the mRNA and protein levels of SAV1, and the ChIP and luciferase assays revealed that KDM2B directly bound to the TSS region of SAV1 and regulated SAV1 expression at the transcriptional level in GC cells. The protein levels of KDM2B and SAV1 in serial GC tissue arrays were then analyzed using IHC. The results showed that KDM2B expression was directly negatively correlated with SAV1. These data revealed that SAV1 was a direct downstream target of KDM2B.

SAV1 is the core component of the Hippo pathway and inhibits the oncogenic transcriptional module (YAP) [7]. Loss of SAV1 induced STAT3 activation and promoted tubulointerstitial fibrosis [25]. In lung cancer, SAV1 could bind to zinc finger protein Gli1 and negatively regulate the Hedgehog signaling pathway [19]. YAP expression was increased in GC, and the Hippo signaling pathway plays a critical role in GC development and progression [6]. Hence, the present study further analyzed the effects of KDM2B on the Hippo signaling pathway in GC. The results showed that KDM2B knockdown led to increased protein levels of SAV1 and phosphorylation of YAP and decreased protein levels of total YAP and its typical downstream target CTGF. These data demonstrated that KDM2B regulated the Hippo pathway via SAV1. However, whether KDM2B further regulates other signaling pathways via SAV1 may be further studied in the future.

In conclusion, the present study provided both clinical and mechanistic evidence, identifying that KDM2B regulated SAV1 expression at the transcriptional level and promoted GC progression. The present study not only identified that KDM2B regulated SAV1 expression but also identified a promising molecular target for new therapeutic strategies for GC.

## Data Availability

All data generated or analyzed during this study are included in this article.

## Supplementary Materials

Supplementary Materials
*Supplementary 1. Supplementary Table 1. Supplementary 2.* Correlation between the clinicopathological characteristics and SAV1 expression (*n* = 100). *Supplementary 3.* Decreased SAV1 expression was positively associated with tumor invasion depth (*T* stage), lymph node metastasis (*N* stage), and TNM stages. *Supplementary 4. Supplementary Table 2. Supplementary 5.* Correlation between the clinicopathological characteristics and KDM2B expression (*n* = 100). *Supplementary 6.* KDM2B expression was positively associated with *T* stages, lymph node metastasis, and higher TNM stages. *Supplementary 7. Supplementary Table 3. Supplementary 8.* Summary of Kaplan–Meier and multivariate Cox regression analysis of overall survival duration in all PADC tissues. *Supplementary 9.* The prognostic value of SAV1/KDM2B and classical clinicopathological characteristics on patient survival was determined by the Kaplan–Meier analysis and log-rank test. Univariate analysis showed that SAV1 expression was positively associated with the OS of patients with GC and that KDM2B expression was negatively associated with the OS of patients with GC. Multivariate analysis showed that age, tumor differentiation, and TNM stages were independent prognostic factors for patients with GC.
