# Supplementary material for: Novel KDM2B/SAV1 Signaling Pathway Promotes the Progression of Gastric Cancer
Source: Genet Res (Camb). 2023 Mar 31;2023:1230182. doi: 10.1155/2023/1230182 (PMC10081913; doi:10.1155/2023/1230182)
Supplement: Supplementary Materials — Supplementary 1. Supplementary Table 1. Supplementary 2. Correlation between the clinicopathological characteristics and SAV1 expression (n = 100). Supplementary 3. Decreased SAV1 expression was positively associated with tumor invasion depth (T stage), lymph node metastasis (N stage), and TNM stages. Supplementary 4. Supplementary Table 2. Supplementary 5. Correlation between the clinicopathological characteristics and KDM2B expression (n = 100). Supplementary 6. KDM2B expression was positively associated with T stages, lymph node metastasis, and higher TNM stages. Supplementary 7. Supplementary Table 3. Supplementary 8. Summary of Kaplan–Meier and multivariate Cox regression analysis of overall survival duration in all PADC tissues. Supplementary 9. The prognostic value of SAV1/KDM2B and classical clinicopathological characteristics on patient survival was determined by the Kaplan–Meier analysis and log-rank test. Univariate analysis showed that SAV1 expression was positively associated with the OS of patients with GC and that KDM2B expression was negatively associated with the OS of patients with GC. Multivariate analysis showed that age, tumor differentiation, and TNM stages were independent prognostic factors for patients with GC. [file 1230182.f1.docx]

Supplementary Table 1

Correlation between the clinicopathologic characteristics and SAV1 expression (*n* = 100)

| Clinicopathological parameters | No.of patients | SAV1 expression (n, %) | | |
| --- | --- | --- | --- | --- |
|  |  | Low | High | *P*-value |
| **Cases** | 100 | 56 (56.0) | 44 (44.0) |  |
| **Age (years)** | | | | |
| ≤65 | 50 | 24(48.0) | 26 (52.0) | 0.149^a^ |
| >65 | 48 | 30 (62.5) | 18 (37.5) |  |
| **Gender** | | | | |
| Male | 36 | 22 (61.1) | 14 (38.9) | 0.440^a^ |
| Female | 64 | 34 (53.1) | 30 (46.9) |  |
| **Tumor differentiation** | | | | |
| Well | 15 | 9 (60.0) | 6 (40.0) | 0.117^b^ |
| Moderate | 74 | 38 (51.4) | 36 (48.6) |  |
| Poor | 11 | 9 (81.8) | 2 (18.2) |  |
| **Invasion depth** | | | | |
| T1+T2 | 15 | 2 (13.3) | 13 (86.7) | <0.001^b*^ |
| T3+T4 | 84 | 53 (63.1) | 31 (36.9) |  |
| **Lymph nodes metastasis** | | | | |
| N0 (negative) | 27 | 8 (29.6) | 19 (70.4) | 0.001^a*^ |
| N1 (positive) | 72 | 47 (65.3) | 25 (34.7) |  |
| **Clinical stages (TNM stages)** | | | | |
| I | 10 | 1 (10.0) | 9 (90.0) | <0.001^b*^ |
| II | 32 | 10 (31.3) | 22 (68.8) |  |
| III | 48 | 36 (75.0) | 12 (25.0) |  |
| IV | 8 | 8 (100.0) | 0 (0.0) |  |

^a^Chi-square test; ^b^Fisher's exact test; ^*^*P* < 0.05 indicates a significant association among the variables.

Supplementary Table 2

Correlation between the clinicopathologic characteristics and KDM2B expression (*n* = 100)

| Clinicopathological parameters | No.of patients | KDM2B expression (n, %) | | |
| --- | --- | --- | --- | --- |
|  |  | Low | High | *P*-value |
| **Cases** | 100 | 45 (45.0) | 55 (55.0) |  |
| **Age (years)** | | | | |
| ≤65 | 50 | 25(50.0) | 25 (50.0) | 0.426^a^ |
| >65 | 48 | 20 (41.7) | 28 (58.3) |  |
| **Gender** | | | | |
| Male | 36 | 16 (44.4) | 20 (55.6) | 0.933^a^ |
| Female | 64 | 29 (45.3) | 35 (54.7) |  |
| **Tumor differentiation** | | | | |
| Well | 15 | 10 (66.7) | 5 (33.3) | 0.050^b^ |
| Moderate | 74 | 33 (44.6) | 41 (55.4) |  |
| Poor | 11 | 2 (18.2) | 9 (81.8) |  |
| **Invasion depth** | | | | |
| T1+T2 | 15 | 13 (86.7) | 2 (13.3) | 0.001^b*^ |
| T3+T4 | 84 | 32 (38.1) | 52 (61.9) |  |
| **Lymph nodes metastasis** | | | | |
| N0 (negative) | 27 | 22 (81.5) | 5 (18.5) | <0.001^a*^ |
| N1 (positive) | 72 | 23 (31.9) | 49 (68.1) |  |
| **Clinical stages (TNM stages)** | | | | |
| I | 10 | 9 (90.0) | 1 (10.0) | <0.001^b*^ |
| II | 32 | 27 (84.4) | 5 (15.6) |  |
| III | 48 | 8 (16.7) | 40 (83.3) |  |
| IV | 8 | 0 (0.0) | 8 (100.0) |  |

^a^Chi-square test; ^b^Fisher's exact test; ^*^*P* < 0.05 indicates a significant association among the variables.

Supplementary Table 3

Summary of Kaplan-Meier and multivariate Cox regression analysis of overall survival duration in all GC tissues

| Clinicopathological parameters | Kaplan-Meier analysis | | Multivariate COX analysis | | |
| --- | --- | --- | --- | --- | --- |
|  | 95% CI | *P*-value | HR | 95% CI | *P*-value |
| **SAV1 expression** | | | | | |
| Low | 18.399-25.601 | 0.008^*^ | 1.139 | 0.621-2.090 | 0.674 |
| High | 29.592-96.408 |  | 1 | - |  |
| **KDM2B expression** | | | | | |
| Low | - | <0.001^*^ | 1 | - | 0.446 |
| High | 16.109-23.891 |  | 1.301 | 0.662-2.558 |  |
| **Age (years)** | | | | | |
| ≤65 | 9.052-76.948 | 0.020^*^ | 1 | - | 0.021^*^ |
| >65 | 4.003-41.997 |  | 1.818 | 1.095-3.020 |  |
| **Gender** | | | | | |
| Male | 16.781-51.219 | 0.839 | 1 | - | 0.729 |
| Female | 0.000-65.280 |  | 1.098 | 0.647-1.864 |  |
| **Tumor differentiation** | | | | | |
| Well | - | 0.002^*^ | 1 | - | 0.024^*^ |
| Moderate | 6.290-43.710 |  | 1 | - |  |
| Poor | 0.000-34.594 |  | 1.925 | 1.089-3.402 |  |
| **Invasion depth** | | | | | |
| T1+T2 | - | 0.011^*^ | 1 | - | 0.917 |
| T3+T4 | 12.974-37.026 |  | 0.951 | 0.373-2.428 |  |
| **Lymph nodes metastasis** | | | | | |
| N0 (negative) | 35.520-84.480 | 0.032^*^ | 1 | - | 0.439 |
| N1 (positive) | 18.330-25.670 |  | 0.712 | 0.301-1.684 |  |
| **Clinical stages (TNM stages)** | | | | | |
| I | - | <0.001^*^ | 1 | - | 0.008^*^ |
| II | 39.871-80.129 |  | 1 | - |  |
| III | 17.641-24.359 |  | 1 | - |  |
| IV | 1.456-12.544 |  | 2.517 | 1.272-4.980 |  |

HR: hazard ratio; 95% CI: 95% confidence interval; ^*^*P* < 0.05 indicates a significant association among the variables.
